# Overview of Vivaldi Antenna Selection for Through-Wall Radar Applications

**DOI:** 10.3390/s24206536

**Published:** 2024-10-10

**Authors:** Mariana Amador, André Rouco, Daniel Albuquerque, Pedro Pinho

**Affiliations:** 1 Departamento de Eletrónica, Telecomunicações e Informática, Instituto de Telecomunicações, Universidade de Aveiro, 3810-193 Aveiro, Portugal; andrerouco@ua.pt (A.R.); ptpinho@ua.pt (P.P.); 2 Escola Superior de Tecnologia e Gestão de Águeda, Instituto de Telecomunicações, 3810-193 Aveiro, Portugal; dfa@ua.pt

**Keywords:** Vivaldi antennas, through-wall radar, ultra-wideband antennas, antenna design and optimization, electromagnetic wave propagation

## Abstract

This paper analyzes broadband antennas, with a special focus on Vivaldi antennas, for their suitability for through-wall radar applications. It assesses various antenna designs, emphasizing high gain, wide impedance bandwidth, and effective wall penetration capabilities. Vivaldi antennas are superior due to their broad bandwidth, high gain, and directional radiation patterns. This study further explores structural optimizations, feeding techniques, and performance enhancement strategies to refine Vivaldi antenna designs for through-wall radar systems. Through a comparative analysis and technical evaluation, this paper highlights Vivaldi antennas’ potential for improving through-wall radar systems’ imaging and sensing capabilities. This presents a pathway for future ultra-wideband advancements.

## 1. Introduction

Through-wall (TW) detection has emerged as a critical research area in response to security challenges, such as asymmetric battlefield threats and domestic defense needs. TW systems have the capacity to enhance situational awareness and effective protection for military applications. On the battlefield, soldiers often face unknown threats hidden behind opaque barriers, requiring strict precaution measures to mitigate risks. Additionally, search and rescue operations and battlefield triage must be conducted with a minimal risk of additional casualties. In this context, technology capable of unobtrusively detecting and monitoring human presence through barriers from a distance becomes an invaluable asset. Traditional optical image sensors are ineffective in visualizing areas behind walls and barriers, leading to the preference for through-wall radar sensors, especially ultra-wideband (UWB) radars. UWB radar systems provide significant advantages in barrier penetration and short-range detection and localization, making them suitable for see-through-the-wall (STTW) applications. Unlike other radar systems, UWB radars have a strong penetrating capability compared to optical systems and offer high-range resolution and superior target discrimination relative to continuous wave (CW) radar systems [1]. By employing impulse or frequency-modulated (FM) signals, UWB radars can measure micro-Doppler movements and differentiate between closely spaced targets. Research has demonstrated that both basic Doppler and UWB radar technologies are effective in STTW applications, particularly due to their barrier penetration strengths. Other technologies, such as millimeter-wave and infrared imaging, provide good resolution through clothing and packaging but face challenges when penetrating higher-density materials like reinforced concrete, concrete blocks, sheetrock, brick, wood, plastic, tile, and fiberglass. These materials can absorb and scatter signals, complicating detection efforts. UWB systems are utilized in various applications, including indoor target localization, tracking, and multi-dimensional imaging. Additionally, advancements in remote biomedical monitoring have highlighted Doppler radar for detecting vital signs as a promising technique for health monitoring and life-sensing applications. This method relies on detecting key features of human periodic motions, and UWB radar is particularly well suited for capturing signals associated with physiological activities such as lung contractions and heartbeats. Through-wall vital sign detection can be divided into single-target and multiple-target detection based on the detection’s complexity. Significant progress has been made in single-target detection, focusing on human respiration, heartbeats, and gait. However, multiple-target detection remains challenging due to issues like low resolution [1,2,3,4,5]. In UWB TW radar systems, antennas play a crucial role, enabling the transmission and reception of electromagnetic waves for imaging and sensing purposes. Ultra-wideband (UWB) is defined as a frequency band greater than 500 MHz or greater that 25% of the operating center frequency. The most common frequency band present in UWB applications is from 3 GHz to 10 GHz, although operating bands from 300 MHz to 20 GHz [6] have started to appear in the literature. Through-wall radar (TWR) systems are included in the family of UWB systems and have gained significant attention in recent years, owing to their ability to detect and obtain images of objects behind walls. One of the main challenges of these systems is that the reflected signal from the wall is stronger than the signal reflected from the human behind the wall. To overcome this challenge there is a need to consider some aspects of the antenna design [7]. To ensure optimal performance in UWB applications, such as TWR, the antenna should exhibit high gain and good impedance matching across the operating bandwidth and operate over a wide bandwidth to achieve high resolution. Additionally, it should be capable of operating in lower frequency ranges for enhanced penetration [7]. Another point to consider in this type of antenna is the size. When lowering the frequency band, the antenna tends to increase in size; therefore, this matter needs to be taken into account. In this article, we discuss different types of UWB antennas in order to know the specifications of each one and determine the most advantageous choice when applied in TWR. There are many different types of UWB antenna designs in the literature; some of them have already been applied in TWR systems [7], others in microwave imaging [8], ground penetrating radars [9] and so on. Antennas can be divided into two major groups: planar and non-planar. Planar antennas can have attractive features such as a low profile, light weight and low cost and they are easy of integration into arrays. These type of antennas have both active and parasitic elements on one plane, making them two-dimensional. They are useful in more discrete situations because of their design. Some examples of planar antennas include the following: Vivaldi, monopole, patch and slot antennas [7]. In terms of planar antennas, the most frequently used in TWR systems is the Vivaldi antenna. This antenna type provides high gain, which allows for the detection of a target at a great depth behind the wall, high directivity (because this type of system suffers from a lot of attenuation, all of the energy needs to be focused on the target), and broadband behavior, therefore exhibiting high-resolution performance, and it also can be used in many forms like Antipodal or Balanced Antipodal Vivaldi antennas. Due to its very lightweight design, this type of antenna can also be turned into array configurations. Another advantage of Vivaldi antennas is that they complement a lot of techniques to enhance their performance. These techniques are based on the feeding mechanism, choice of substrate, shape of flare, etc. Although there is a long way to go in understanding the behavior of Vivaldi antennas and how can we miniaturize them without damaging their performance at lower frequencies, there are already some studies reviewing this techniques [10]. Further in this article, we will also present some descriptions of them in order to understand the working method of this antenna type. Patch-like antennas are also already applied in TWR [11], and when comparing these antennas to the Vivaldi antennas, Vivaldi antennas can obtain a wider bandwidth and better resolution [7]. In terms of the non-planar antennas, these antennas do not lie in a single plane; instead, they are deposited on a curved substrate. These types of antennas have a major drawback compared to planar antennas: their manufacture can be more difficult, which leads to higher costs [12]. Horn antennas are prominent in the literature when talking about non-planar antennas applied in TWR applications [13,14]. These antennas are used due to their great directivity, small side lobes, high gain and their UWB behavior. The drawback of this type of antenna compared to the Vivaldi one is in terms of volume. Horn antennas are considered bulky and big, which makes them harder to install and transport. In this article, we present a comparison between UWB antennas to prove the use of Vivaldi antennas as the best choice for TWR systems. We also present a deep study of this antenna type and the performance enhancement techniques that can be applied on Vivaldi antennas and their results.

This paper is divided into six sections. Section 1 summarizes UWB antenna types, the requirements for TWR system antennas and what is studied in this paper. In Section 2, we discuss and analyze types of UWB antennas and their pros and cons when applied to TWR systems. In Section 3, the authors discuss and conclude the best antenna choice to apply in TWR systems. In Section 4, the working principle of Vivaldi antennas is analyzed and we study the feeding structure of the antenna. Section 5 presents the most used techniques applied on Vivaldi, Antipodal Vivaldi and Balanced Antipodal Vivaldi antennas in order to enhance the performance of the antenna, from the gain to the reflection coefficient values. To finish, Section 6 summarizes the conclusions on everything studied in this paper.

## 2. Types of UWB Antennas

In this section, we present a review of several UWB antenna designs. It starts with the discussion of Vivaldi antenna design characteristics and the types of Vivaldi antennas such as Antipodal Vivaldi and Balanced Antipodal Vivaldi. Planar monopole and slot antennas are also discussed; although they do not present a directive behavior that is needed in TWR systems, these antenna types are present in microwave imaging applications or in ground-penetrating radar systems [9]. The last planar antenna discussed is the bow-tie antenna. In terms of non-planar antenna designs, the horn and the dielectric resonator antennas will be discussed. In Table 1, we present a comparison of related works for UWB antennas.

### 2.1. Vivaldi Antennas

The Vivaldi antenna is a type of end-fire traveling-wave antenna that is highly suitable for applications requiring a broad bandwidth and directional characteristics. The core design of Vivaldi antennas includes an exponentially tapered planar slot, typically fed through electromagnetic coupling by means of a microstrip-to-slotline transition. This antenna operates with a non-resonant radiation mechanism, where waves travel along the tapered slot, confined between the conductors. As the slot widens, the energy is dispersed into space through the radiating slot [34]. Vivaldi antennas are capable of achieving a fractional bandwidth of up to 100% and provide a gain ranging from 5 to 8 dBi [35,36]. They can also achieve a fidelity factor of 90% [37]. These antennas are utilized in various fields, including microwave imaging systems for biomedical purposes and through-wall radar applications.

#### 2.1.1. Antipodal Vivaldi Antenna (AVA)

**Figure 1:**
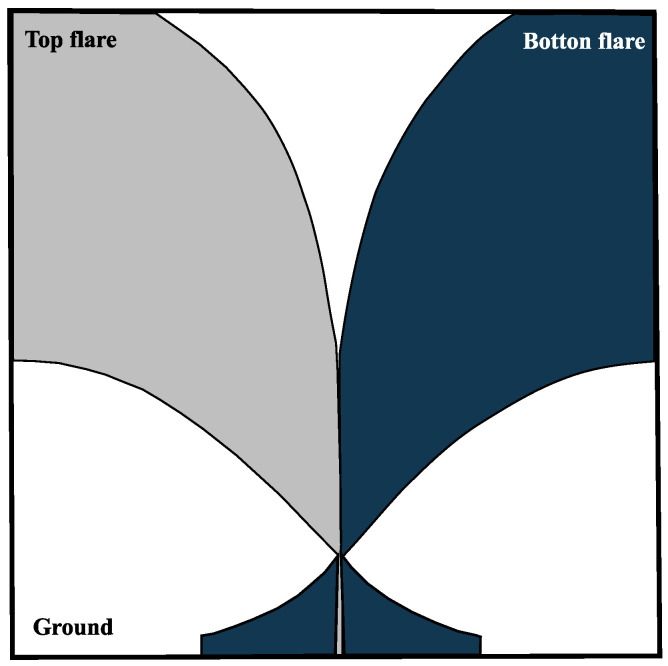
Structure of an Antipodal Vivaldi antenna.

The design of an Antipodal Vivaldi antenna (AVA) is derived from traditional Vivaldi antennas, with modifications made to the radiator section. In this configuration, the two arms of the radiator are positioned on opposite sides of the substrate. This arrangement, combined with its unique feeding mechanism, enhances the bandwidth of the standard Vivaldi antenna. The feeding structure of AVA involves a transition from a microstrip line to a double-sided parallel stripline, which facilitates low-frequency operation. The inner and outer edges of the radiator arms are shaped with exponential curves, as described in [38]. Figure 1 illustrates the AVA’s structure, where the top flare functions as the signal conductor and the bottom flare serves as the ground plane, with both flares being symmetrical to each other [39].

The Antipodal Vivaldi antenna has been used in several studies in comparison to the conventional Vivaldi antenna due to its advantages, such as its high gain, high efficiency, low return loss, reduced side lobe levels, high-frequency operation, and wide-bandwidth capabilities. This antenna also provides a stable radiation pattern. In Table 2, we present some of the advantages of AVA design.

#### 2.1.2. Balanced Antipodal Vivaldi Antenna (BAVA)

This type of Vivaldi antenna consists of three copper layers: The outer two copper layers are connected to the feed line and act as the ground. The middle layer of the antenna is connected to the signal layer and acts as a conductor. All copper layers are separated by substrates and their structures can be seen in [50]. Owing to its particular structure, the antenna is capable of balancing the loading of the dielectric material between the conductor and ground plane. One of the advantages of this design is that the change in the direction of the beam due to the frequency and polarization, also known as beam quint, is reduced, owing to this balance [50,51]. BAVA is able to achieve better gain, a reduced level of cross-polarization and a wide bandwidth when compared to the conventional AVA but has some major disadvantages such as its design complexity and fabrication cost, which leads to AVA design or even the conventional Vivaldi design being preferred, with several enhancement performance techniques being applied on it to achieve the desired results [52].

### 2.2. Planar Monopole Antennas

Planar monopole antennas are a type of antenna that has gained significant attention in radar applications due to their simple structure, wideband characteristics, and compact size. These antennas are characterized by their single conductor (monopole) placed over a ground plane, typically printed on a planar dielectric substrate. Their wideband performance makes them particularly suitable for various radar systems, such as ground-penetrating radar and microwave imaging systems, where a wide range of frequencies are necessary for high-resolution detection and imaging. The compact and low-profile design of planar monopole antennas allows them to be easily integrated into modern radar devices, which often require portability or must fit within limited spaces. This makes them ideal for applications in environments where space and weight constraints are critical, such as in automotive radar and portable surveillance equipment. Additionally, their good radiation efficiency ensures that a significant portion of the transmitted power is effectively radiated, which enhances the radar’s ability to detect and obtain images of targets accurately. Planar monopole antennas typically have an omnidirectional radiation pattern, providing broad coverage, which is advantageous for applications like perimeter surveillance and search and rescue operations. Their simple planar structure also allows for their easy integration with other electronic components on printed circuit boards (PCBs), making them versatile and cost-effective for modern radar systems that often combine multiple functionalities in a single compact unit [53,54]. Planar monopole antennas feature a radiating element that can be fed either through a microstrip line or a coplanar waveguide (CPW).The ground plane on the backside of the antenna can be either fully or partially covered. A full ground plane provides the antenna with directional radiation properties, which can be beneficial in some radar applications. However, this configuration typically results in a narrower bandwidth, which might limit its effectiveness in applications requiring wideband capabilities. On the other hand, a partial ground plane can support a wider-bandwidth operation, making it more suitable for applications that require the antenna to operate efficiently across a broader range of frequencies. This flexibility in design and feeding techniques further enhances the adaptability of planar monopole antennas for various radar applications. With this type of antenna, it is possible to achieve a fractional bandwidth of 100% and a typical fidelity factor of about 75%. The gain that is possible to achieve is about 5 dBi, but using the typical ground system, the gain is about 2 to 3 dBi [55]. There are some techniques consisting of a perturbation of the ground plane that are applied to planar monopole antennas to enhance the bandwidth. In [56], the authors introduce an elliptical planar monopole antenna optimized for radar applications. The antenna design focuses on enhancing its bandwidth and radiation efficiency, which are vital for radar systems that require reliable target detection and imaging. The elliptical shape helps to achieve a broader bandwidth and improved impedance matching. The study demonstrates the antenna’s effectiveness in radar applications where space constraints and high performance are necessary, such as in unmanned aerial vehicles (UAVs) and portable surveillance systems. The antenna’s design allows for flexibility in various radar applications, making it a versatile choice for different operational environments. The study in [57] presents two ultra-wideband (UWB) planar monopole antennas with elliptical shapes, primarily developed for applications such as breast cancer detection. These antenna designs are notable for their configuration of ground planes and the dimensions of their elliptical patches, which significantly impact their performance. Each antenna features an elliptical-shaped patch radiator on the top side, fed by a 50 Ω microstrip line, while the bottom side is equipped with a partial ground plane incorporating different slot arrangements. In radar applications, similar design principles can be applied to optimize antennas for specific detection and imaging tasks. For example, the careful selection of ground plane configurations and patch dimensions can enhance the antenna’s bandwidth and radiation characteristics, making it more effective for detecting various targets. In this work, the first antenna design includes rectangular slots oriented at a $45^{\circ }$ angle, while the second design has slots positioned at $90^{\circ }$. These configurations influence the antenna’s operational bandwidth, allowing it to cover frequencies from 3 to 20 GHz, which is advantageous for high-resolution radar imaging and detection across various environments. A CPW feed quasi-cross slot-based circular monopole antenna was proposed in [58]. The antenna provides a wide bandwidth from 3.1 to 10.6 GHz. Another UWB antenna study is present in [59]. In this study, the authors developed two antennas with two half-heart shapes to monitor cardiac activities. The two antennas are printed in different substrates to achieve different dimensions. The antenna has similar behavior to a Vivaldi antenna in terms of horizontal direction radiation. The microstrip line is curved to facilitate the connection with the SMA connector. These antennas have a frequency bandwidth from 3.1 to 10.6 GHz. The gain obtained is between 7.4 and 8.81 dBi with $45^{\circ }$ oriented slots. For a $90^{\circ }$ oriented slot, a gain ranging from 7.15 to 9.08 dBi along the frequency band of 3 to 20 GHz is obtained. In terms of planar monopole antennas, their size can be reduced by making slots in the ground plane or using parasitic elements. A compact wideband planar monopole antenna with a defected ground structure (DGS) is developed in [54]. This study focuses on a compact wideband planar monopole antenna with a DGS optimized for modern radar sensing systems. The defected ground structure is used to enhance the antenna’s bandwidth and radiation characteristics. The paper demonstrates how this antenna design achieves a broad frequency range while maintaining a compact form factor, making it suitable for portable radar systems used in applications like ground-penetrating radar. The DGS improves impedance matching and reduces surface wave effects, which is crucial for high-resolution radar imaging applications. In [60], a square monopole antenna for microwave imaging is presented, with the dimensions of 10mm×10mm. By making four cuts in the corner and also utilizing an inverted U-shaped parasitic element, a frequency band of 3.8 GHz to more than 9 GHz is obtained. This antenna also provides a fidelity factor of 90%. Another technique to provide a wider impedance bandwidth is to cut an open-circuit structure in the ground plane of the monopole antenna and insert an inverted pi-shaped parasitic structure in the ground plane [17]. This modification causes additional resonances at 11.5 GHz and 14.2 GHz. A similar technique is used in [18]. In this antenna design, a rotated E-shaped slot was made in the ground plane and an E-shaped parasitic structure was designed behind the feed line that consisted of a microstrip line. This method provided an extension of the upper frequency limit from 10.3 GHz to 15 GHz. The dimensions of the antenna were 12 mm × 18 mm. In [9], a UWB monopole antenna for ground-penetrating radar application was designed. This antenna has a size of 143 mm × 125 mm and yields a −10 dB impedance bandwidth from 0.5 to 6.5 GHz. A very compact size monopole antenna is present in [61], with the dimensions of 9 mm × 10 mm. This antenna was designed for microwave imaging systems. The design of the antenna consists of a monopole printed on the upper face of the substrate and a ground plane on the bottom face. They both have a quarter of an ellipse shape. In order to improve the antenna ’s performance, in the radiated and ground plane, pairs of symmetrical slots were added, which increased the current patch close to the termination of the radiating arms of the antenna. The results showed a bandwidth from 2.1 to 11 GHz. The time domain of the antenna is also analyzed and the authors obtain a noticeable distortion on the receive pulse, which makes this antenna suitable for medical imaging applications. In [62], a monopole planar antenna is proposed with a size of 10.2 mm × 15.5 mm and with a frequency range from 4.23 GHz to more than 14 GHz. The antenna design incorporates a radiating patch, a feed network, and a partial ground plane. The radiating element is constructed using four identical metamaterial unit cells, which are combined to achieve a wide bandwidth and maintain a compact antenna size. A linear tapered microstrip line is employed to feed the radiating patch, enhancing impedance matching across the operational frequency range. The antenna demonstrates gain values that range from 2 to 5.17 dBi, indicating its effectiveness across various frequencies.

It can be noted that the majority of monopole planar antennas use low permittivity substrates to achieve a more compact size, and most of these antennas are able to achieve a fractional bandwidth higher than 110%. Because of its omnidirectional radiation pattern, this type of antenna is not frequently used in radar applications, and when it is used, it is for ground penetration radar applications since others may require more directivity characteristics.

### 2.3. Planar Slot Antennas

Planar slot antennas consist of a narrow slot etched on the ground plane with a microstrip feed line printed on the other side of the substrate. The microstrip conductor is short-circuited through the dielectric substrate with the longer side of the slot [63]. Good impedance matching and a wide impedance bandwidth can be achieved with this configuration by properly terminating the open end of the feed line. Center-feed [63] or offset-feed [64] methods are used to excite the radiation slot, with the center-feed type having a lower impedance bandwidth compared to the offset feed. In order to improve the radiation characteristics, a reflector or a cavity can be added to the back side of the design, although this increases the complexity of the antenna’s structure. Several techniques are used to enhance the antenna bandwidth. In [65], a CPW-fed elliptical slot antenna with a tuning uneven U-shaped stub on a flexible liquid crystal polymer is proposed for tumor detection. This antenna is able to achieve a bandwidth from 1 to 12 GHz. A CPW-fed wideband slot antenna was proposed in [23], where the antenna was excited by a tapered monopole, and an arc-shaped slot was adopted to increase the operating frequency band, achieving a frequency range from 2.89 to 12.58 GHz by positioning a tuning stub inside the radiating slot. In [22], a compact and planar antenna with a double-elliptical slot and differential feed is presented for medical diagnosis applications. This antenna has a reduced size of 25 mm × 36 mm. Two elliptical-shaped radiators are design on the top part of the substrate, and a double slot-based ground is designed on the bottom part. Two tapered microstrip lines with a phase difference of $180^{\circ }$ feed the double elliptical slots. To achieve a compact antenna capable of operating in the frequency range from 1 to 9 GHz, a differential feed is integrated into the antenna, also providing a constant gain above 8 dBi in the frequency range from 2 to 8 GHz. In [66], a compact antenna (23 mm × 21 mm) is designed for breast tumor detection. The radiating part consists of a modified patch with circular and rectangular shapes and a ground plane consisting of the tapered slot. Both are printed on the FR-4 substrate. The antenna has an average gain of 4.1 dBi in the frequency band from 3.1 to 12 GHz. The average efficiency of the proposed antenna is above of 90%, with a maximum of 96%. The fidelity factor, representing the cross-correlation value between the transmitted and received signal, is 87% and 91% in face-to-face and side-by-side configurations, respectively. Planar slot antennas could be suitable for radar applications due to their potential to provide a wide bandwidth, good impedance matching, and high radiation efficiency, which are important for detecting and imaging targets across various frequencies. Their compact size and low profile also offer advantages, making them potentially ideal for integration into portable and space-constrained radar systems. Moreover, their ability to achieve directional radiation patterns may allow them to focus energy more effectively on specific areas.

### 2.4. Bow-Tie Antennas

The bow-tie antenna features two triangular conductive elements printed on a circuit board, with both elements being fed at their narrow ends, commonly referred to as the bow apex [67]. This antenna is essentially a planar version of the traditional biconical antenna. In this antenna type, the abrupt termination of the current distribution at the edges of the triangular elements can lead to some bandwidth limitations. However, several techniques can be employed to enhance the performance and extend the bandwidth of the antenna. For example, in [24], a planar bow-tie slot antenna was developed to increase the bandwidth of the conventional bow-tie antenna to 40%. The study introduced a tapered metal stub at the center of the bow-tie slot, which further enhanced the impedance bandwidth and introduced a new resonant frequency at a higher band, significantly improving overall bandwidth performance. Another study in [68] presented a novel bow-tie antenna with a pyramidal shape designed for radar imaging applications. The antenna’s radiating structure consists of a pair of horn-shaped, tapered bow-tie arms on a dielectric substrate with a slotline between the arms to improve impedance matching. To achieve a more directional radiation pattern, a horn-shaped pyramidal reflector was added to the back of the bow-tie arms. Comparisons between the antenna with and without the reflector showed that the antenna with the reflector exhibited superior unidirectional radiation characteristics, making it suitable for high-directive radar imaging applications. This design operates within the frequency range of 3.06 to 11.7 GHz and achieves a peak gain of 8.5 dBi. Additionally, in [69], a compact bow-tie antenna optimized for ground-penetrating radar (GPR) applications is proposed. This antenna features a resistive loading technique using a thin sheet of graphite to achieve ultra-wideband (UWB) performance and minimize end-fire reflections. The modified design includes rounding the sharp corners of the antenna arms, which improves impedance matching and broadens the operational bandwidth from 0.4 GHz to 4.8 GHz. The simulation results show that this design provides enhanced radiation efficiency, increased gain, and reduced reflections, making it highly effective for GPR applications where clear signal detection and minimal interference are crucial. These studies collectively demonstrate the versatility of planar bow-tie antennas in radar applications. The ongoing advancements in antenna design, such as the incorporation of resistive loading and optimized feed structures, continue to improve the effectiveness of bow-tie antennas in various radar technologies, including ground-penetrating radar and radar imaging systems.

### 2.5. Horn Antennas

Horn antennas have a wide range of applications, from small-aperture to large-aperture antennas used to feed reflectors. These antennas can be excited in any polarization or a combination of polarizations. Horn antennas come in various forms, with the most common shapes being pyramidal and conical [70]. This type of antennas does not have a specific radiating element, allowing it to operate over a wide range of frequencies while exhibiting high gain values that can reach 20 dBi or more. In [30], a transverse electromagnetic (TEM)-mode horn antenna is designed to operate over a frequency range from 2 to 12 GHz. To match the impedance of the coaxial connector, a microstrip-to-parallel strip balun was developed for this antenna. The results show a VSWR of less than −31 dB over the frequency range from 2 to 13 GHz. Horn antennas are generally known to be large and bulky, but there are studies focused on techniques to miniaturize these antennas. In [29], a compact ceramic double-ridge horn antenna was proposed for biomedical UWB radar applications. The design used a metal ceramic material with a permittivity of 70, resulting in an overall aperture size of 16 mm × 11 mm. The frequency range of the proposed antenna is from 1.5 to 5 GHz, with a gain of 8 dBi within the operating frequency range. The use of a high dielectric constant material affects the input impedance of the antenna, reducing it to the value of 15 Ω. To address this issue, a UWB power amplifier was integrated into the antenna. The amplifier’s function is to match the low input impedance of the antenna to the standard impedance of the equipment, ensuring optimal performance. In summary, horn antennas are essential designs for radar applications due to their high gain, wide bandwidth, and flexibility in polarization. While traditionally large and bulky, ongoing research aims to miniaturize these antennas and enhance their performance through innovative designs, using, for example, high dielectric materials and integrating UWB power amplifiers. These advancements make horn antennas versatile and effective for both conventional radar systems and emerging applications, such as biomedical imaging and compact ground-penetrating radar.

### 2.6. Dielectric Resonator Antennas

A dielectric resonator antenna (DRA) is composed of dielectric material with high permittivity, such as ceramic material, mounted on a perfect ground plan or on a grounded dielectric substrate with lower permittivity [71]. To enhance the radiation characteristic of DRAs, the design can be integrated with metal reflectors, dielectric lenses, and other components. In [72], a circularly polarized DRA design is present using a low-cost, lossy FR4 epoxy substrate and thick FR4-based DRA. This design is excited by using four-line feed with a circular loop-type network. The loop, with its cross-shaped arms, introduces a $90^{\circ }$ phase difference to to achieve circular polarization. The optimized antenna has an overall size of 30 mm × 30 mm and operates in the frequency range from 24 to 27 GHz providing a constant gain of 8.6 dBi within this band. Another study [31] presents a compact cubical dielectric resonator antenna for radar-based microwave imaging. This antenna achieves a peak gain of 5.97 dBi and operates over a frequency rage from 4.3 to 12.6 GHz. The results also show a measured gain of 3.88 dBi between 4.31 and 6.4 GHz and an average of 4.09 dBi from 9.3 to 13.2 GHz. While DRAs can be compact, they generally offer a lower fractional bandwidth value.

## 3. Discussion: The Choice of Vivaldi Antenna

In Section 2, various types of antennas with broadband capabilities suitable for different applications were presented. In Table 3, we present a comparison of antenna designs applied in though-wall radar systems. Now, we can discuss the most suitable antenna for radar-through-wall (TWR) applications. Vivaldi antennas are particularly advantageous due to their ability to provide a wide impedance bandwidth and high gain. While some designs of Vivaldi antennas are relatively large, research has focused on miniaturization techniques to reduce their size. Vivaldi antennas can operate at lower frequencies, down to 500 MHz [73]. However, a key drawback when operating at these lower frequencies is a decrease in gain and an increase in size. Furthermore, when examining UWB Vivaldi antennas at lower frequencies, there is a noticeable reduction in the frequency range. Planar monopole antennas, although highly compact, offer a wide frequency band with gains between 5 and 10 dBi. Nevertheless, their radiation pattern is generally not suitable for TWR applications because they lack the required directive behavior. Slot antennas, like monopole antennas, offer a compact size, wide impedance bandwidth, and acceptable gain. However, bow-tie antennas typically exhibit a narrower bandwidth than required for TWR applications, though their operating frequency starting at 500 MHz ensures effective penetration through objects. Horn antennas are another type commonly used in radar applications due to their large impedance bandwidth and high directivity. However, they are not compact and have higher production costs. Dielectric resonator antennas (DRAs) provide a compact design, but their bandwidth is not as favorable compared to other antenna types [72]. Archimedean planar spiral antennas are designed for broadband applications, including those that require coverage over low-frequency bands. These antennas have a spiral-shaped conductive element that expands outward from a central point, providing a continuous path for current flow, which supports a wide range of frequencies. Unlike some compact antenna designs, Archimedean planar spiral antennas can be relatively large because their size is related to the number of turns in the spiral and the spacing between them. However, they are still considered compact relative to their ability to cover a broad spectrum, making them suitable for applications requiring a wide bandwidth, such as ultra-wideband (UWB) radar systems. Their size and form factor can be optimized for specific applications, but they might not be as compact as some other antenna types when designed for very low-frequency operations. Among all the antennas discussed, Vivaldi and horn antennas are the most commonly used in TWR applications due to their wide bandwidth and high directivity. To be capable of selecting antennas for through-wall radar (TWR) applications, several key characteristics must be considered to ensure their optimal performance. Table 4 outlines the essential features that make an antenna suitable for TWR applications. These characteristics directly affect the radar’s ability to detect and image objects through obstacles such as walls.

Given these characteristics, Vivaldi antennas stand out as the optimal choice for UWB applications in through-wall radar due to their high gain and UWB behavior, which results in better resolution and enhanced target differentiation. Additionally, Vivaldi antennas are lightweight and easy to install. They can be employed in various forms, such as antipodal or exponentially tapered Vivaldi antennas, and can function as single antennas or in an array configuration. To achieve optimal performance, various characteristics of Vivaldi antennas must be optimized, including feeding and performance enhancement techniques.

## 4. Overview on Vivaldi Antennas

**Figure 2:**
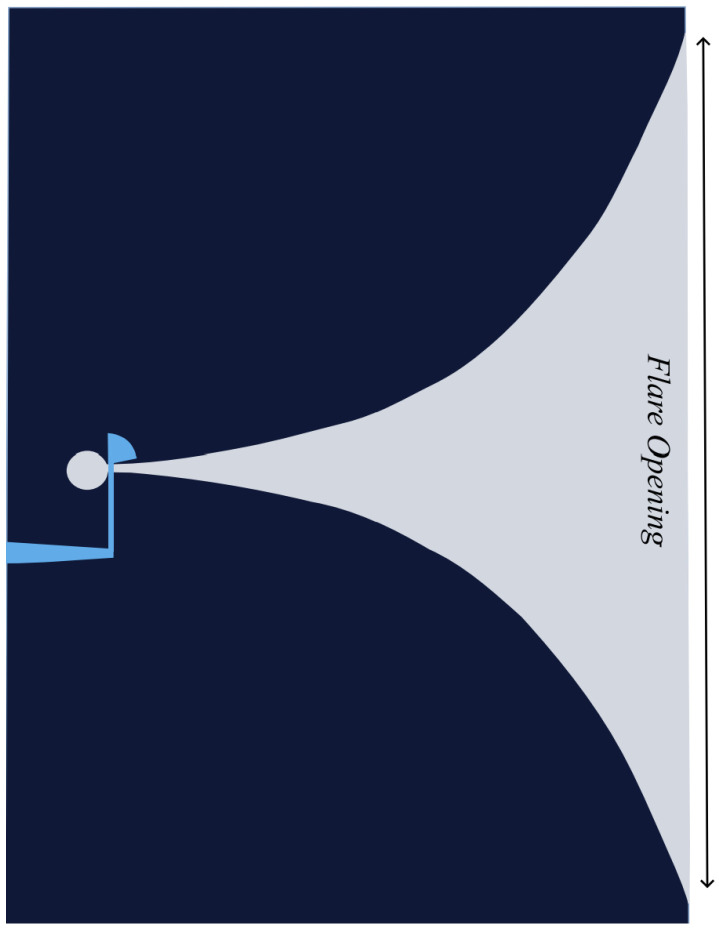
Structure of a Vivaldi antenna.

The Vivaldi antenna, Figure 2, belongs to the category of periodically structured, continuously scaled, gradually curved, and slow leaky end-fire traveling-wave antennas. In different segments of the antenna, various frequencies radiate, though the radiating portion maintains a constant size relative to the wavelength. Theoretically, Vivaldi antennas can operate over an infinite range of frequencies with a stable beam width. However, in practice, their bandwidth is constrained, often due to transitions within the feeding structure, such as the shift from a transmission line to a slotline, which can reduce the available bandwidth [38]. Two key design challenges for a Vivaldi antenna are creating a feeding structure that supports a wide frequency range with minimal reflection and determining the dimensions and shape of the antenna to achieve a focused beam. To better understand its operation, the antenna’s surface can be divided into two sections: the propagating region and the radiating region. The slot width, or the distance between the conductors, is less than half the wavelength in free space (λ0/2) in the propagating section, keeping the traveling waves bound to the conductors. As the slot width expands to more than half a wavelength, the bond diminishes, allowing the energy to radiate into the surrounding air. The waves travel along the antenna until they reach the point where their phase velocity matches the speed of light in free space. This limit case is typical for antennas using air as the dielectric material [34]. It can be inferred that low-dielectric substrates play a significant role in optimizing the radiation and overall performance of the antenna. Vivaldi antennas, with their wide operating bandwidth and end-fire radiation pattern, have proven essential in applications such as radar through-wall imaging. Their ability to maintain a constant beam width across a broad frequency range, combined with low reflection from the feed transition, makes them an ideal choice for high-performance radar systems, particularly in environments where penetrating solid obstacles is critical.

### Feeding Mechanisms

**Figure 3:**
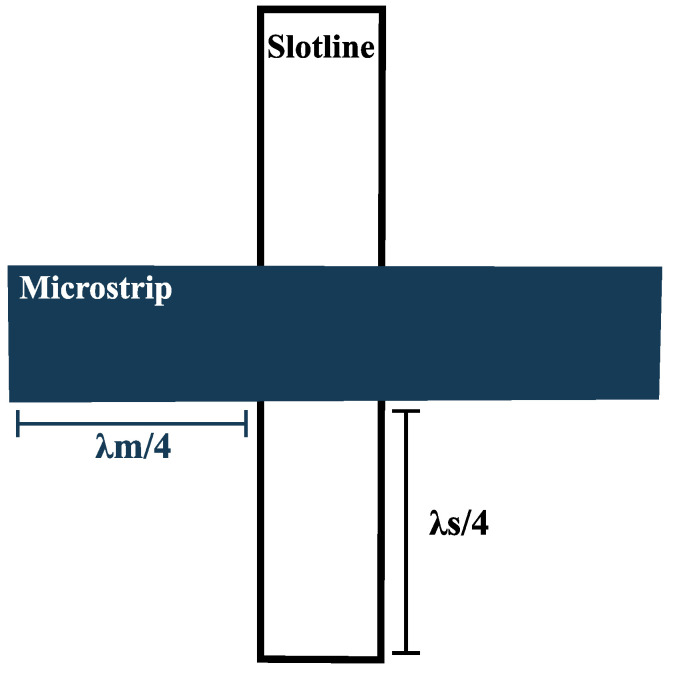
Geometry of a simple microstrip-to-slotline transition.

In Vivaldi antennas, the feed line facilitates power transfer between the source and the antenna. Typically, the characteristic impedance of the transmission line is 50 Ω. According to the maximum power transfer theorem, to achieve optimal input power, the Vivaldi antenna should be fed at a point where its input impedance is also 50 Ω. As part of the tapered slot antenna family, the Vivaldi antenna is most efficiently fed through a carefully designed transition that couples signals to a slotline, ensuring low-loss transmission over a wide frequency range. There are two primary feeding methods used for this purpose. The first is the directly coupled transition, which uses a wire or solder connector to establish an electrical connection. One common example of this method is the coaxial line to slotline transition. The second method involves electromagnetically coupled transitions, where signals are transferred to the slotline via electromagnetic fields. An example of this approach is the microstrip-to-slotline transition, where a microstrip on one side of the substrate crosses a slotline on the opposite side at a specific angle. The microstrip and slotline each extend a quarter wavelength beyond the crossing point. In this configuration, the open-circuited microstrip appears as a short circuit, while the slotline, grounded on the opposite side, behaves as an open circuit when crossing the microstrip [79]. Figure 3 illustrates a basic microstrip-to-slotline transition. One major limitation of this type of transition is its significant reduction in bandwidth. To address this issue, Schieck and Koler [80] proposed a six-port microstrip-to-slotline transition, though this solution proved to be complex and difficult to implement.

**Figure 4:**
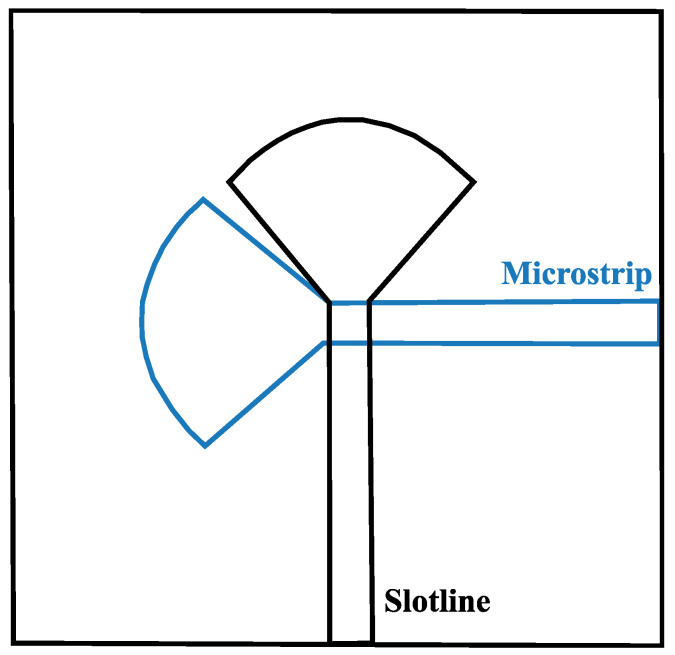
Structure of a radial stub.

Antipodal slotline transition also belongs to electromagnetically coupled transitions. This transition was proposed to overcome the mentioned disadvantages; moreover, it offers a lower impedance compared to the slotline. The input part of this type of transition is a microtrip. The paired-strip corresponds to the transition region and the slotline is the radiating section. Despite the improvements in the bandwidth, this type of transition has poor cross-polarization characteristics. The Balanced Antipodal Vivaldi antenna (BAVA) was developed to correct some of the issues of the Antipodal Vivaldi antenna (AVA) such as its poor cross-polarization characteristics. So, the feeding mechanism of the balanced antipodal slotline is also included in the electromagnetically coupled transition, and it is based on an additional dielectric and metal layer balancing the E-Field distribution in the flared slot, improving the cross-polarization. The antennas that used this type of feed are fed directly by a stripline [79]. To match the perfect conditions related to the feeding techniques, there are a lot of research studies that test various types of feeding for Vivaldi antennas. In reference [81], a tapered microstrip feed line with a fixed port width of 1.5 mm is employed to achieve ideal impedance matching. In [82], the feeding mechanism utilizes a transition from a broadside parallel stripline to a coplanar waveguide, allowing for easier integration into radio frequency (RF) circuits. This design is characterized by a wide operational bandwidth and minimal insertion loss. Similarly, in [83], a compact, tapered microstrip feed with a bending structure is used to ensure wide impedance matching. This feed is composed of two metallic bends positioned on both sides of the substrate. In [84], an ultra-wideband (UWB) antipodal tapered slot antenna is designed using elliptical strip conductors. The feeding mechanism for this antenna incorporates an elliptically shaped ground plane, a microstrip line, and a parallel plate, creating a balun transformer for balanced signal transmission. In reference [81], a tapered microstrip feed line with a fixed port width of 1.5 mm is employed to achieve ideal impedance matching. In [82], the feeding mechanism utilizes a transition from a broadside parallel stripline to a coplanar waveguide, allowing for easier integration into radio frequency (RF) circuits. This design is characterized by a wide operational bandwidth and minimal insertion loss. Similarly, in [83], a compact, tapered microstrip feed with a bending structure is used to ensure wide impedance matching. This feed is composed of two metallic bends positioned on both sides of the substrate. In [84], an ultra-wideband (UWB) antipodal tapered slot antenna is designed using elliptical strip conductors. The feeding mechanism for this antenna incorporates an elliptically shaped ground plane, a microstrip line, and a parallel plate, creating a balun transformer for balanced signal transmission. In [85], a Vivaldi antenna is excited using a microstrip-to-slotline transition balun, connected to an SMA connector, which provides broadband impedance matching. A similar feeding technique is described in [15], where an exponentially tapered microstrip line is used to excite a fern-shaped fractal antenna. The transition between the feed line and the radiator, with an exponential taper, influences the impedance bandwidth. In [86], a microstrip feed is aligned with the exponential flares of a corrugated Vivaldi antenna, ending in a radial open stub to enhance input impedance bandwidth. Another conventional Vivaldi antenna design in [87] utilizes a microstrip feed with a width of 1.14 mm to match the 50 Ω coaxial line, optimizing bandwidth for detecting voids in concrete specimens. In [46], a compact Antipodal Vivaldi antenna is driven by a simple coaxial feed for wideband applications. Meanwhile, a two-stage coupled bandpass filter with a K-type connector is implemented in the feed structure of an antenna in [88], providing low insertion loss, proper impedance matching, and an adequate bandwidth. In [89], a stepped connection between the slotline and tapered patches on the back plane of the antenna significantly improves impedance matching and the bandwidth. A highly directional Vivaldi antenna is introduced in [90], featuring a feed line and three metal patches for impedance transformation from 50 Ω to about 80 Ω. This antenna’s geometry includes a circular taper and slotline for wideband operation. A three-port diversity antenna design for vehicular communication is described in [91], using three independent Vivaldi antennas on a single printed circuit board to produce a wide radiation pattern and high antenna gain. This system incorporates a conventional Antipodal Vivaldi antenna fed by a 50 Ω coaxial cable, with two additional planar Vivaldi antennas fed by microstrip lines. In [92], a balun consisting of a T-junction power divider and two different transition structures—microstrip to CPW and microstrip to coplanar stripline—is used to excite a Vivaldi antenna, achieving balanced phase and magnitude over a wide bandwidth. A compact UWB balun design for printed balanced antennas is presented in [93], where a combination of transitions from microstrip to slotline and electromagnetic coupling ensures wideband performance. The antenna in [94] uses two orthogonal Vivaldi elements with electromagnetic coupling between the microstrip and slotline, improving the design by reducing its size and simplifying assembly. Lastly, in [95], a compact end-fire Antipodal Vivaldi antenna for UWB radar and wireless systems is proposed, employing a bending feed line and sinusoidal tapered slot for compactness. See Figure 4.

## 5. Techniques Used for Performance Enhancement of Vivaldi Antennas

To make Vivaldi antennas more compact while maintaining their performance within the required specifications, various methods have been proposed, such as optimizing the substrate material, modifying the flare shape, incorporating slots, and using advanced feeding techniques. These methods are designed to enhance the electrical and physical properties of the antennas, improving bandwidth, radiation patterns, size, and other key performance parameters. While many enhancement techniques have been developed and studied [96], there is still a lack of comprehensive focus on their specific applications in ultra-wideband (UWB) radar systems, particularly in through-wall scenarios. In radar applications, especially UWB radar, the compact design of Vivaldi antennas is crucial for maintaining high performance in terms of signal penetration and resolution. However, a further exploration of how these enhancement techniques can be tailored to the specific needs of UWB radar, such as improving target detection and imaging through obstacles like walls, remains an important area for continued research and development.

### 5.1. Substrate Material

The choice of substrate material is a critical factor in antenna design, particularly given the wide range of substrates with varying relative permittivity. To enhance antenna performance, a substrate with low loss tangent (δ), low relative permittivity (ϵr), and adequate thickness is desirable. Lower relative permittivity reduces the antenna’s size at a given resonant frequency by increasing the fringing field and aperture area, which can improve radiation characteristics. The loss tangent indicates the power lost within the substrate material itself; a lower loss tangent leads to lower power loss and, consequently, higher gain and efficiency. A substrate with high relative permittivity tends to reduce both the electric field and the antenna’s gain, which is undesirable for high-performance applications. In ultra-wideband (UWB) radar, particularly for through-wall imaging, these substrate characteristics are critical for balancing size and performance. Since UWB radar systems require a broad bandwidth, low power loss, and stable radiation patterns, substrate selection plays a significant role in ensuring the antenna performs effectively under demanding conditions. For example, in through-wall radar applications, a thicker substrate with low permittivity can improve signal penetration and enhance the radar’s ability to detect and image objects behind barriers. Common substrates used in Antipodal Vivaldi antennas include FR-4, Rogers RO4003, and Rogers RT/Duroid 5880. In [97], a compact AVA was fabricated on an FR-4 substrate, with a thickness of 1.6 mm, a permittivity of 4.4, and a loss tangent of 0.02. The antenna operated in the frequency range of 3.6 GHz to over 12 GHz, achieving a peak gain of 4.5 dBi. In [43], an antenna built on Rogers RO4003 with a thickness of 0.508 mm, a permittivity of 3.38, and a loss tangent of 0.0027 operated from 3.4 GHz to 40 GHz, achieving a peak gain of around 15 dBi and a return loss of −50 dB. The antenna in [98] used a Rogers RT/Duroid 5880 substrate and achieved a peak gain of 8.5 dBi across a frequency range of 0.67 to 6 GHz with a return loss of −42 dB. In [99], a comparison between FR-4 and Rogers RO3006 substrates revealed that the antenna designed with RO3006, with a permittivity of 6.15, loss tangent of 0.002, and thickness of 1 mm, offered better compactness and return loss (−40 dB) compared to the FR-4-based design. The FR-4 antenna had a permittivity of 4.4, loss tangent of 0.02, and thickness of 1.4 mm, yielding a return loss of −28 dB and a slightly lower peak gain of 5 dBi. In conclusion, while different substrates such as Rogers RO4003, RT/Duroid 5880, and RO3006 offer distinct advantages in terms of bandwidth, gain, and return loss, their application in UWB radar systems, especially for through-wall imaging, highlights the importance of substrate choice. Substrates with low permittivity and loss tangent values not only improve the overall antenna performance but also allow for better signal penetration and imaging capabilities, which are crucial for radar systems operating in complex environments.

### 5.2. Flare Shape

The shape of a Vivaldi antenna’s flare is crucial for its radiation performance. The flare shape determines the aperture area, and as this area increases, so does the radiation, leading to improved antenna performance, including higher gain. However, the challenge lies in achieving higher radiation without increasing the aperture area, as this would result in a larger antenna. A narrower flare can make the antenna more compact but at the cost of reduced performance, particularly in terms of gain. To address this issue, many researchers have proposed various shapes, such as circular, elliptical, and leaf-inspired designs. The elliptical shape has a easy design, meaning this shape is the most common in Antipodal Vivaldi antennas. In the elliptical shape flare present in [100], we observe straight lines between the two end-points, and upon extending these lines, we obtain the circular shape that is present in [101]. This circular shape creates the same radiation pattern in E and H planes, improves impedance matching [102] and increases the frequency band [101]. In [15], the design of the Vivaldi antenna is based on a fern leaf-inspired fractal structure. The antenna is an Antipodal Vivaldi-type antenna, radiating maximally in the end-fire direction with less directivity at lower frequencies, and the leaf flare shape will improve the cross-polarization of the AVA. The drawback of this antenna is reflect on the complexity of the design. In [103], the antenna design is based on a Chebyshev flare shape. Using this shape, the frequency bandwidth can be improved. This flare shape shows good impedance matching at a lower frequency with a stable radiation pattern in almost all of the frequency ranges. Other designs are still being tested like bow-tie flare and windmill flare shape [104]. These shapes are more simple to design and still give a compact size to the antenna. The drawback in these two cases is the decrease in the antenna’s gain. In Table 5, we present a comparison of various flare shapes and their major results. It is noticeable that an elliptical shape, besides being easier to implement, results in a bigger antenna size.

### 5.3. Slots and Corrugations

Slots are regions where metal is removed from the flares of an antenna, and when these slots are evenly spaced along the edge of the flares, the structure is referred to as corrugations. Slots play a critical role in enhancing antenna performance, particularly with regard to bandwidth. For example, in [106], an Exponential Slot Edge Antipodal Vivaldi Antenna (ESE-AVA) is introduced, where the slots help extend the lower bandwidth limit, reduce side lobe levels, and increase the main lobe level. Similarly, in [107], linear slots are incorporated into a conventional AVA design to prevent unwanted backward radiation. In this design, both the top and bottom conductors are corrugated with three linear slots to improve overall performance. In [108], circular slots of different diameters are applied to the flares of an AVA, which not only improve the antenna’s impedance characteristics but also enhance its radar cross-polarization (RCP) by minimizing orthogonal radiation. This results in a compact design with dimensions of 32 mm × 35 mm × 1.6 mm, though it achieves a moderate peak gain of 5.5 dBi. One approach to achieving compactness in tapered slot antennas is the inclusion of L-shaped slots along the radiating fins, which increases the electrical length of the antenna and lowers the operating frequency while maintaining the same physical dimensions. However, this modification can lead to radiation from the outer edges of the antenna, so the slot dimensions must be carefully selected to ensure the antenna’s resonance remains stable [83]. Additionally, in [109], symmetrical slots are introduced to reduce the width of the antenna without compromising its radiation pattern, Figure 4 in [109]. In the obtained results, it can be seen that adding slots caused the elimination of the unwanted currents that radiate vertically with the end-fire direction marked by the region A. In region B, we can observe significant currents along the slot edges indicating that the effective length of the current path on the antenna is lengthened through the modification. This comparison between the current distribution of an original AVA and a tapered slot edge AVA at 3.5 GHz demonstrates the effectiveness of these design modifications. The estimate lengths of the slot are obtain using Equation (1), presented below, for the lowest and highest frequency of the frequency bandwidth.

(1) $$l_{\mathrm{corrugation}}=\frac{\left(\frac{\lambda_{r}}{\sqrt{\epsilon_{r}}}\right)\left(\sqrt{\frac{1\;+\;\epsilon_{r}}{2}}\right)}{4}$$

To obtain the length of the other slots, an individual optimization is conducted until we obtain the desired impedance matching. To choose the locations of the slots, an analysis of the current distribution of the antenna with and without the slots present is conducted as in [110]. The square selected as (a) presents the current distribution without the slots, the square selected as (b) presents the current distribution at 9 GHz in the corrugated Vivaldi antenna, and the square selected as (c) presents the current distribution at 20 GHz, and a plot with a comparison of the gain of the antenna with and without the slots is also presented. It can be observed that in the Vivaldi antenna without the corrugations, the current is distributed along the outer edges of the antenna. Adding the slots will introduce additional resonances by increasing the electrical length. Each slot will be responsible for a resonance at a distinct frequency, therefore redistributing the current around itself. It is also noticeable that for the largest wavelengths that correspond to lower frequencies, the current is concentrated on the largest slots and the opposite happens for the smaller wavelengths that make the current concentrate in the shortest slots. With all the modifications, it is concluded that the incorporation of the slots can increase the gain of the antenna, improve the impedance bandwidth and reduce the cross-polarization level. Other research studies have been using the addition of slots in Vivaldi antennas to obtain an enhancement in their performance. In [111], an Antipodal Vivaldi antenna with UWB performance is developed. The authors of the study made the modifications by steps, starting with a conventional AVA (CAVA). Then, they extended the frequency band by bending the inner edges of the top and bottom radiators of the conventional antenna. Finally, to increase the gain at the lower frequencies, they applied regular triangle splits in the antenna. The final antenna design has low cross-polarization, less than −15 dB at the entire frequency range of 2 to 27 GHz, and a lower cutoff frequency and has the capability of improving the front-to-back-ratio in comparison to the AVA with just bending the outer edges. There are also researchers that apply the slots to a Balanced Antipodal Vivaldi antenna. In [112], a BAVA is proposed with a frequency bandwidth from 6 to 18 GHz. The antenna design consists of three copper layers, where two of the layers are external and located on the two outside areas of the dielectric substrates. To the conventional BAVA, the authors include an extra supporting part of the substrate beyond its aperture. This extra part of the substrate with three copper layers is further cut in a triangular shape. An I-shaped slot-loaded radiation patch is also used. This modifications can be seen in Figure 3 of [112]. It is also possible to identify through the current distribution that without the slots, there is a concentration of the current on the edge of the antenna. When the slots are applied, the concentration moves to the aperture of the antenna, which leads to the obtained results exhibiting a good impedance bandwidth and an improvement in the end-fire performance, also being able to achieve high gain in the end-fire direction, low cross-polarization and a high front-to-back ratio. It is also possible to use multiple periodic slots at different locations with different widths and lengths. In [113], an AVA is designed with a compact size of 40 mm × 90 mm × 0.508 mm. The authors start adding a slit-edge technique to the conventional AVA, creating a periodic slit-edge Antipodal Vivaldi antenna (PSEAVA). Then, to the PSEAVA, they add a trapezoid-shaped lens (TDL) as an extension of the substrate used to optimize and increase gain and directivity. The use of a dielectric lens will be explained further. The addition of the slots attempts to decrease surface current intensity on the edges of the top and bottom radiators, leading to an extension of the low end of the frequency band, increasing the gain and improving the front-to-back ratio at lower frequencies. The results obtained for this antenna are very satisfying compared to other AVA antennas. The authors obtain a return loss of −45 dB, a gain from 6 to 15 dBi and a frequency range from 3.4 to 40 GHz. In [114], the effect of the sizing of the corrugations is presented. The authors develop three antenna designs. Design A has a uniform depth of rectangular corrugation. Design B has a depth of corrugation that decreases linearly. On the opposite side, design C increases the corrugations linearly. The results obtained show that, in design C, we have a marked decrease in the gain as the frequency increases. In designs A and B, we obtain a return loss below −15 dB over the entire operating frequency band. In design B, we obtain the best gain level of 16.2 dBi, and the next best gain level is from design A with 15 dBi. For design B, a low cross-polarization level of −22 dB compared to −15 dB of designs A and C is obtained. In [114], the electric field distribution at the aperture of the antenna for all three designs is also analyzed. The perfect way to obtain the best performance is via an electric field horizontally distributed. What we can see from the results obtained is that designs A and C show some degradation in the electrical field shown in the presented circles. On the other hand, design B, where the corrugations decrease linearly, shows no degraded distribution, obtaining a completely horizontal electric field. For this reason, design B improved the side lobe levels in the E and H planes. In [115], an Antipodal Fermi tapered slot antenna is designed. This TSA is based on the “FERMI antenna” proposed by Sugawara [116]. This antenna has a profile defined by the Fermi–Dirac function, and it was found that the Fermi antenna has almost the same E and H plane patterns and low side lobe levels. The authors opted to use a sine type of corrugation pattern and curved tapered ends that, when used together, improve the radiation characteristic. Sine corrugation is preferred compared, for example, to a rectangular shape with shaped edges because it reduces the effects of current disruption on the shaped angles and eliminates anti-phase waves in the aperture. Another Fermi tapered slot antenna with sine corrugation is developed in [117]. In this research study, the authors conduct a detailed analysis on the sine corrugation shape compared to no corrugation and rectangular corrugation. This comparison is shown in Figure 2 of [117]. It can be noticed that the Vivaldi with no corrugation shows a larger anti-phase wave region and a highly reflected wave at the feeding structure. The rectangular-shaped corrugation decreases the reflected wave between the slots in the region marked as C. The antenna with the sine-shaped corrugation also creates a more balanced current distribution. Another possibility of slots is comb-shaped slots on an elliptical flare seen in [49]. The study also presents the results of the gain enhancement and extension of the operating frequency band at lower frequencies. In the context of ultra-wideband (UWB) radar, particularly for through-wall imaging, the use of slots in Vivaldi antennas is highly beneficial. By incorporating slots, the antenna’s bandwidth is extended, enabling more effective penetration through solid barriers such as walls. Furthermore, the improved radiation pattern and reduced side lobe levels achieved through slot implementation allow for better target resolution and imaging quality in radar systems. For example, corrugated slot designs can help minimize interference from unwanted reflections, improving the clarity of radar images when detecting objects behind walls. These performance enhancements make slot-based Vivaldi antennas a good choice for UWB radar applications, where both a broad bandwidth and precise radiation control are essential for accurate through-wall detection and imaging.

### 5.4. Dielectric Lens

A dielectric lens functions as a director and is typically placed between the two flares at the end of an antenna’s aperture. The dielectric lens should be a shaped object with high permittivity, acting as a waveguide to direct most of the energy toward the center of the aperture. The phase velocity in the lens will be lower than in the rest of the substrate structure, creating differences in the propagation velocities between the lens and the copper edges. This results in a faster wave traveling along the edges, and since the path length is longer, the overall effect is a more planar phase front near the antenna’s aperture. When designing the dielectric lens, its shape should be optimized to avoid reflections from its extremities [50]. Various dielectric lens shapes are discussed in the literature, some of which are shown in Table 6.

In [50], a Balanced Antipodal Vivaldi antenna for a breast cancer detection system is proposed. The antenna directivity is improved by the inclusion of a director and an analysis of the antenna with and without the dielectric lens is conducted. The results of the reflection coefficients show that there is no significant change in the working bandwidth. On the other side, the transmission coefficient ($S_{21}$) shows that the BAVA with a dielectric lens increases the $S_{21}$ magnitude. An antenna with higher $S_{21}$ receives more reflected energy from an object placed in front of the antenna. When examining the radiated field, a clear distinction can be seen between the two antennas depicted in Figure 11 of [50]. The radiated E-field is shown for both the standard BAVA and the BAVA equipped with a dielectric lens (BAVA-D). The end-fire radiation pattern is noticeable, and it is apparent that the director greatly improves the near-field directivity of the BAVA. However, it is noticed that there is an increase in the back radiation. The front-to-back ratio maintains 35% higher compared to the conventional BAVA. The radiation behavior through the frequency domain can also be analyzed. It is observed that the effect of the dielectric lens increases as the frequency also increases [50]. In both [47,87], Vivaldi antennas with half-elliptical-shaped dielectric lenses are presented. In [47], the authors design a Balanced Antipodal Vivaldi antenna with further implementation of the dielectric lens. A comparison of the best size of the half-elliptical dielectric that is shown in [47] is conducted. Upon analyzing the given graph, it is concluded that the best size for parameter b in the dielectric lens is 50 mm because a higher size of the director will result in a very long extension of the antenna, which will complicate the practical applications of the antenna [47]. The half-elliptical dielectric lens is also applied in an Antipodal Vivaldi antenna in [87] and the gain enhancement using the director compared to the miniaturized conventional AVA is also visible. A rod shape is used in [118]. Dielectric rods are made with a vast array of different materials, but when using high dielectric constant materials, they can result in a decrease in the cross-sectional area. The antenna presented has low insertion loss, high mutual decoupling efficiency and a broadband input match. It also provides a frequency-independent radiation pattern. A new trapezoidal shape is adopted in order to decrease the energy that scatters away from the aperture when using a half-elliptical-shaped dielectric. The new shape achieves a higher gain in the boresight direction and concentrates most of the energy at the aperture center of the antenna, as can be seen in Figure 4 of [43]. A parametric analysis on the length of the dielectric lens is also conducted. The conclusion taken is that the size of 30 mm is the most adequate length since this is when a high gain value is achieved. Another study [119] also suggests that the trapezoidal dielectric lens increases the gain to more than 10 dB. The use of the director also provides good directional radiation with a well-formed main beam across the frequency band. The trapezoidal shape can also eliminate the pattern asymmetry of the conventional AVA, which solves the problem of gain peak shifting and improves the main-axis gain of the antenna [120]. In [48], the authors present an AVA with a high-permittivity dielectric lens consisting of two fan-shaped planar dielectrics with different permittivity values and a half-circular-shaped planar dielectric lens with the same permittivity as the AVA’s substrate. A parametric study on the permittivity values was conducted and the authors analyzed the gain. This study also proves the enhancement in radiation performance when using a dielectric lens, which also obtained a higher gain. Dielectric lenses are present in the correction of main beams that can be tilted away from the axis. In [121], the authors present a parametric study on the length of a triangular-shaped dielectric lens that shows that the E-plane beam tilt is decreased by an increase in the length of the dielectric lens. Because it is more suitable for practical applications, when increasing the length of the triangular-shaped director, the end of the substrate is truncated, resulting in a flat-end shape. Conducting an overall analysis, the highest gain of 16.5 dBi is achieved in [119] with the trapezoidal-shaped dielectric lens. However, the highest return loss of −52 dB is achieved in [48] using the circular-shaped dielectric lens.

### 5.5. Parasitic Patch

A parasitic patch in Vivaldi antennas refers to an additional conductive element that is not directly connected to the feed line. This patch is positioned between the two flares of the antenna and helps to focus the radiation beam more effectively in the end-fire direction. By incorporating a parasitic patch, the antenna’s directivity is improved without negatively affecting its performance at lower frequencies, and it also reduces the need for electrically thin dielectric substrates, offering more flexibility in design [122]. In ultra-wideband (UWB) radar applications, particularly in through-wall radar systems, parasitic patches play a crucial role; by concentrating the radiated energy in a specific direction, the patches enhance the antenna’s ability to penetrate obstacles like walls, improving target detection and imaging accuracy. The focused beam produced by the parasitic patch ensures better signal direction and efficiency, which is vital in UWB radar systems where precise imaging and high resolution are required. Similar to dielectric lenses, parasitic patches can come in various shapes, as shown in Table 7, allowing for further optimization based on the specific needs of radar systems.

In [122], the authors present an Antipodal Vivaldi antenna with an elliptical parasitic patch introduced in the flare aperture of the antenna. The purpose was to decrease the performance degradation at higher frequencies, without compromising the frequency range. The reason for the patch producing a stronger radiation in the end-fire direction is because instead of having the radiation production only from the fields coupled between the arms, at high frequencies, the fields also couple to both sides of the parasitic patch, limiting the off-axis radiation that stems from the phase reversal issue [122]. Similar to what was stated by Nassar in [122], the position where the parasitic patch should be placed is between the two flares of the antenna to improve the coupling. A Vivaldi antenna including first an elliptical parasitic patch was proposed and further replaced by a elongated elliptical parasitic patch that can be seen in Figure 1 of the article [88]. The proposed parasitic shape provides better coupling, obtaining a higher gain of 8.48 dBi. The shape is also modified to improve the radiation performance. Slits in the edges and a bandpass filter in the feed are also added in order to increase the control of the end-fire radiation pattern and the sub-harmonic suppression [88]. A trapezoidal parasitic patch is designed in [123] to radiate the maximum part of the beam in the end-fire direction by coupling with the main radiating patch. This will overcome the radiation degradation at higher frequencies. The proposed antenna has a peak gain higher than 9 dBi in the frequency band from 6 to 26.5 GHz. The reasons stated in the article for the trapezoidal parasitic shape are the following: to ensure that the field is coupled to both sides of the parasitic element and to transform E-Field distribution into a plane wave form. For this, the right trapezoid is designed in order that the sides have a decreased profile toward the end-fire direction. As a result of adding the parasitic element, the antenna shows a stable radiation pattern over the frequency band without any complex structures [123]. A circular parasitic patch with a circular dielectric lens is implemented in an Antipodal Vivaldi antenna to make a compact and high-gain antenna for UWB purposes [46]. A comparison of the peak gains at different frequencies is conducted and the results show that at 3 GHz, the AVA with a lens and the AVA with a lens and dielectric have a similar gain of 5.2 dBi compared to the 4.7 dBi gain of the conventional AVA. At 6.5 GHz, the results change and the worst peak gain goes to the AVA with a lens and dielectric with 3.5 dBi compared to the almost 4 dBi gain of the AVA with a lens and the 3.96 dBi gain of the conventional AVA. The major difference in and benefits of the parasitic patch happens at 10.6 GHz and the peak gain obtained is 6.7 dBi compared to the 3.86 dBi peak gain of the AVA with just the dielectric lens and the 4 dBi peak gain of the conventional AVA. These results are also proved by the current distribution shown in Figure 3 in [46]. It is noticeable that the current in the parasitic patch in the lower frequencies is weak and the patch has almost no influence on the antenna’s performance. On the opposite side, at higher frequencies, the patch is responsible for increasing the gain. The use of a diamond-shaped parasitic patch is adopted in [124] for a wideband end-fire conformal Vivaldi antenna array mounted on a dielectric cone. This diamond patch is designed to also improved the gain in the upper frequency as seen in the other studies. Different shapes were analyzed in different articles for an improvement in the antenna’s performance. The elliptical shape is the overall preferred shape because it divides the tapered slot aperture into two smaller tapered apertures that produce radiation in the end-fire direction and avoids sharp corners that can affect the radiation pattern.

### 5.6. Metamaterial

Metamaterials or double-negative materials are artificial structures that offer electric and magnetic properties that are not found in nature. These materials have negative permittivity and negative permeability at the same frequency. Their properties are provided from the structure and not the material that they are made of. Usually, they are used in antenna designs in order to obtain a UWB bandwidth, high gain, and electrically small structures. Metamaterials can be used in antenna substrates, in the direct antenna geometry or as a lens [125]. Usually, these materials are designed manually with the help of natural materials. In [39], the authors propose a compact AVA with improved directivity using zero-index metamaterials with a bandwidth from 1.3 GHz to 12 GHz. The results achieved are a maximum gain of 12.4 dBi, with both E and H planes’ beam widths almost being equal. The metamaterial unit used consists of a meander-line resonator printed on an FR4 substrate. This resonator will work as a band-stop filter at the frequency of 9.3 GHz. With the results from the analysis, it is possible to conclude that the metamaterial units do not disturb the return loss of the antenna, keeping its ultra-wide property, but they also do not improve this factor. On the other side, a 4 dBi gain enhancement compared to the conventional AVA with a noticeable side lobe level reduction is obtained. This technique will also correct one of the drawbacks of Vivaldi antennas, which is their low gain at lower frequencies [39]. In [42], the authors use epsilon-near-zero metamaterial unit cells that are applied in the flare aperture of the Vivaldi antenna. These unit cells confine the radiated fields with an associated compact size. In this case, the metamaterial is composed of periodic patches printed on a dielectric slab with a periodicity that is much smaller than the operating wavelength. To control the equivalent dielectric permittivity, it is necessary to adjust the shape of the patches and the space between them. In this case, the epsilon-near-zero material has only one component of the permittivity tensor close to zero. A snug-in negative-index metamaterial is implemented to increase the performance of an AVA in [126]. Seven layers of metamaterial surfaces are inserted at the center and at a right angle of an AVA to enhance the gain compared to just one single layer of the metamaterial. It was shown by the results that a multi-layered negative-index material AVA performs better at increasing the gain. It is proved that using metamaterial technology has a positive increase in performance for practical applications such as UWB, see-though-wall radar imaging [126]. By tailoring the electromagnetic properties of the antenna, such as gain and directivity, metamaterials help improve signal penetration through barriers like walls, allowing for more accurate detection and imaging. Their ability to focus and confine radiated fields makes them ideal for UWB radar systems, providing better resolution and higher efficiency, especially in challenging environments. As a result, metamaterials offer a promising avenue for advancing the capabilities of radar systems in applications like through-wall imaging and other forms of non-invasive detection.

### 5.7. Optimization Algorithms and Machine Learning

The fast simulation of antenna designs on computer platforms has opened opportunities for optimizations to facilitate the design processes and help in a variety of engineering electromagnetic problems. An evolutionary algorithm was proposed based on particle swarm optimization (PSO) and was introduced into the electromagnetic (EM) community. Its applications have received enormous attention in recent years due to its functionalities [127]. Besides PSO, computational intelligence uses other techniques like multi-objective PSO (MOPSO) and a multi-objective genetic algorithm (MOGA). These techniques can be used to decide the dimensions and optimize the different parameters of the antenna. In [128], the authors present an Antipodal Vivaldi antenna with the objective of reducing three parameters: transient distortion, the reflection coefficient and the cross-polarization level. To achieve the objectives imposed, a multi-objective particle swarm optimization (MOPSO) implemented in MATLAB is used in order to handle all three requirements at once. The optimization of the UWB AVA was achieved, with transient distortion and the cross-polarization level minimized. A time-modulated antenna array using UWB Vivaldi antennas is proposed in [129]. The array is composed by seven elements and works in a bandwidth range from 2.65 GHz to 17.26 GHz. In order to achieve a low side-lobe level and side band level, the particle swarm optimization technique is used. The results of the simulations showed a good performance with the proposed antenna array. A compact BAVA with a size of 32 mm × 35 mm × 1.6 mm is designed using the conformal finite difference time domain (CFDTD) technique with the particle swarm optimization algorithm [130]. The advantage of using computational algorithms is that we can train them based on the requirements of the application being used, and it is also possible to focus the optimization in some of the requirements. They also have some disadvantages like consuming more time, and the results may be drastically different if the training carried out for the wanted requirements is not conducted correctly, and because of this, more interactions may be required in order to have the optimum solution, which ends up consuming more time compared to other antenna enhancement performances already mentioned before.

Additionally, one popular approach in machine learning is the use of supervised learning, where a dataset of antenna designs and their corresponding performance metrics are used to train a model. Once trained, the model can predict the performance of new antenna designs without the need for full-wave electromagnetic simulations. For instance, deep learning models have been used to optimize the geometry of ultra-wideband (UWB) Vivaldi antennas, which are often employed in through-wall radar systems. These models can predict the impact of small geometrical changes on bandwidth and radiation patterns, improving design efficiency. Another way of using machine learning is applying a convolutional neural network (CNN) trained using a dataset of UWB antenna designs and their corresponding features. The CNN could predict the parameters in real time, significantly speeding up the design process. This approach is particularly useful in optimizing the antenna’s ability to produce clear, high-resolution images in through-wall radar applications [131]. Machine learning has opened new possibilities for optimizing antennas in through-wall radar systems, providing more efficient, faster, and accurate methods for improving antenna performance. From neural networks predicting design outcomes to genetic algorithms searching for optimal configurations, ML-based approaches significantly reduce the design cycle time and improve the ability of TWR antennas to penetrate various materials. As the field of antenna design continues to evolve, the integration of machine learning techniques will likely play an increasingly important role in advancing radar systems for applications like security, search and rescue, and non-invasive monitoring.

### 5.8. Discussion

Some of the most known techniques were present and explained in terms of major contributions in antenna performance. It is concluded that the substrate choice can influence the gain, bandwidth, return loss, efficiency and size of the antenna. The addition of slots and corrugations is mainly focused on influencing the bandwidth, side and main lobe of an antenna, the return loss and also the co-polarization level. The flare shape choice of the antenna can have impact on size, bandwidth and return loss. Parasitic patches and dielectric lenses are techniques used to improve the gain, radiation pattern and directivity. The use of metamaterial is also known for producing an increment in gain, the possibility of size reduction and a improvement in the return loss and bandwidth of the antenna. Additionally, Vivaldi antenna arrays have significant potential for enhancing the performance of through-wall radar applications due to their high gain, wide bandwidth, and directional radiation patterns. In TWR systems, where signal penetration and imaging accuracy are crucial, arrays of Vivaldi antennas can provide stronger and more focused signals, improving detection behind obstacles like walls. The array configuration allows for better control over beam steering and resolution, which is essential in radar imaging. However, the main challenge is the increased physical size and complexity of such arrays, which must be managed carefully to maintain the system’s portability and practicality. Studies, such as [132,133], have explored the application of Vivaldi arrays, including in TWR. Despite the benefits, designing Vivaldi arrays for TWR requires optimizing the trade-offs between enhanced performance and system size, especially for applications where compactness is important. In Table 8, we present examples of Vivaldi antennas with broadband behavior, with some of the discussed enhancement performance techniques applied.

## 6. Conclusions

This article unites several pieces of information on UWB antennas in order to conclude what the best choice of antenna for TWR systems is. It starts with a deep study and comparison of different broadband antennas, their characteristics and major applications. When comparing Vivaldi, monopole, slot, bow-tie, spiral and horn antennas, it was stated that the Vivaldi antenna was the one with more advantages for the requirements needed. This antenna present us with a good UWB and directivity behavior that is needed in TWR to achieve the high resolution needed and also to be able to detect a target in great depth behind a wall. The other valuable point of this antenna type is its discrete structure and easy construction and design. Further on in this paper, a study was conducted on the working principle of Vivaldi antennas to be able to understand some results obtained in different articles. We also presented a study of the substrate impact on the antenna, including comparisons between feeding structures and performance enhancement techniques, from slots to dielectric lens, applied on this antenna. In terms of the most recent applications on the Vivaldi antenna, we also present some examples of computational intelligence and metamaterial applications with successful results, which proves the capacity of this antenna to be able to achieve an even better performance. The Vivaldi antenna has a lot potential still to be understood and developed but has already shown us its advantages for use in TWR systems.

## Figures and Tables

**Table 1:** Comparison of related work on UWB antenna designs.

Reference	Date	Antenna Type	Frequency [GHz]	Gain [dBi]	Size [${\mathrm{mm}}^{2}$]
[15]	2017	Vivaldi	1.3–20	10	50.8×62
[1]	2016	Vivaldi	0.85–9.55	6	–
[16]	2017	Vivaldi	2–16.3	11	54.7×75
[17]	2012	Planar monopole	2.91–14.72	–	12×18
[18]	2014	Planar monopole	2.95–14.27	6	12×18
[19]	2016	Planar monopole	3.1–10.6	9.4	50×85
[20]	2014	Planar monopole	2.96–15.8	6.1	12×18
[21]	2021	Planar monopole	7–28	10.1	50×60
[22]	2013	Planar slot	1–9	12	25×36
[23]	2009	Planar slot	2.89–12.58	6	64×63
[11]	2015	Planar Patch-like	0.5–2	–	140×170
[24]	2004	Bow-tie	8–16.8	8	18.85×11.62
[25]	2018	Bow-tie	0.98–4.5	10.3	107.7×68
[26]	2023	Bow-tie	0.5–5.5	4.9	100×100
[27]	2021	Bow-tie dipole	0.5–1.5	–	–
[28]	2019	Horn	2–4	–	–
[29]	2013	Horn	1.5–5	8	16×11
[30]	2010	Horn	2–12	–	–
[31]	2020	DRA	4.3–12.6	4.09	15×20
[32]	2016	Archimedean planar spiral	2.15–2.75	–	–
[33]	2022	Archimedean spiral	1.7–7.5	2	65×65

**Table 2:** Advantages of Antipodal Vivaldi antenna.

Advantage	Details	Reference
Gain	Antipodal Vivaldi antenna and its array can provide higher gain above 18 dB.	[40,41]
Return Loss	AVA is able to achieve an improved return loss up to −50 dB.	[42,43]
Efficiency	AVA efficiency goes up to 90%. Because of its improvement in return loss, the antenna also obtains good impedance matching.	[44]
SideLobe Level	AVA design reduces the side lobe level below −13 dB.	[45]
Compact size	AVA design can be present with various miniaturization techniques in order to have a compact size.	[46]
Radiation Pattern	The radiation pattern of the AVA is stable, symmetric and not frequency dependent.	[47]
Front-to-Back-Ratio	AVA design can maintain front-to-back-ratio at a high level.	[48,49]

**Table 3:** Comparison of antenna designs applied in through-wall radar applications.

Reference	Date	Antenna Type	Frequency	Type of Walls	Target Detection
[32]	2016	Archimedean Planar Spiral	2.15–2.75	Concrete: 30 cm	Distinguish Fan Motion Frequency from Human Vital Signals
[74]	2019	Vivaldi	2–4	Wooden: 9 cm	Vital Signs
[27]	2019	Bow-Tie Dipole	0.25–0.75	Thick Brick: 28 cm	Classification between Humans and Dogs
[75]	2019	Bow-Tie	–	Brick and Concrete: 20 cm	Vital Signs
[28]	2019	Horn	2–4	Plastered Brick: 13.5 cm and Drywall: 6.25 cm	Human Motion
[76]	2021	Bow-Tie	0.5–1.5	Two-Layer Brick	Vital Signs
[77]	2022	Vivaldi	center Frequency: 5	Thick Wooden Board: 4.2 cm and Concrete: 28 cm	Vital Signs and Motion Detection
[78]	2022	Vivaldi	0.5–2.5	Double-Layer Concrete brick: 23 cm	Human Motion

**Table 4:** Key characteristics of antennas for TWR applications.

Characteristic	Reason	Benefit
Wide Bandwidth	To achieve high resolution and sufficient penetration depth.	Enables high-resolution imaging, which is critical for detecting small objects behind walls and provides flexibility to adapt to different materials and environmental conditions.
High Gain	To focus energy in a specific direction, increasing the effective range and penetration capability.	Enhances the ability to detect weak signals from behind thick or dense walls.
Directivity	Helps concentrate the beam in the desired direction and reduces the effects of multipath interference from walls and objects.	Improves the signal-to-noise ratio, providing clearer images and reducing false detections.
Compact and Lightweight Design	Portability is crucial for rescue, tactical operations, and field applications where quick and efficient movement is required. A compact design is also easier to integrate into other systems.	Greater mobility and ease of use in various operational settings and reduces the physical burden on operations.
Low Cross-Polarization	Improves the antenna’s ability to reject unwanted signals, such as clutter and noise, from other directions.	Increases detection accuracy and performance in more complex indoor environments by minimizing interference from unwanted signals.

**Table 5:** Comparison results between different flare shapes.

Reference	Flare Shape	Substrate	Size (${\mathrm{mm}}^{3}$)	Frequency [GHz]	Return Loss [dB]	Gain [dBi]
[101]	Circular	RT5880	64×64×2.54	4–50	−30	3–12
[100]	Elliptical	FR4	100×172.14×1.6	1–6	−40	−1–6
[15]	Leaf	FR4	50.8×62×0.8	1.3–20	−35	10
[103]	Chebyshev	FR4	100×100×1.6	1–35	−52	4.5–6.8
[105]	Bow-Tie	FR4	26×31×1.6	2.8–3.4; 4.2–5.1; 6.1–11.6	−31	0–4.2

**Table 6:** Comparison of the results between different dielectric lens shapes.

Reference	Director Shape	Substrate	Size (${\mathrm{mm}}^{3}$)	Frequency [GHz]	Return Loss [dB]	Gain [dBi]
[50]	Diamond	RT6002	78×44×9.2	2.2–12	−40	–
[47]	Half-Elliptical	AD255	96×50×3.15	3–18	−27	5–14.7
[87]	Half-Elliptical	RO4003	96×100×0.508	1–30	−40	2–12
[118]	Rod	RO4003	100×90×1.524	7–13	−36	14.5
[119]	Trapezoidal	RO4003	60×40×1	3.68–50	−45	2–16.5
[120]	Trapezoidal	RO4003C	57.5×60.7×0.508	3.3–40	-	3.8–12.6
[43]	Trapezoidal	RO4003C	40×60×0.508	3.4–40	−50	8–14
[48]	Circular	RT5880	171.8×149.8×1	1.3–30	−52	1–16.5
[121]	Triangular	–	76×56×1.5	6–20	-	9.4–12

**Table 7:** Comparison between different parasitic patch shapes.

Reference	Parasitic Patch Shape	Substrate	Size (${\mathrm{mm}}^{3}$)	Frequency [GHz]	Return Loss [dB]	Gain [dBi]
[122]	Elliptical	RT6002	140×66×1.5	7–32	−27	0–12
[123]	Trapezoidal	TLC-30	124×66×1.575	6–26.5	−32	3–12
[46]	Circular	FR4	34×16×0.8	3.01–10.6	−30	3.27–6.7
[124]	Diamond	Teflon F4B	124×116×0.5	2.2–12	−50	2.5–9.8

**Table 8:** Comparison of related works for broadband Vivaldi antennas.

Reference	Date	Antenna Technique	Frequency [GHz]	Gain [dBi]	Size [${\mathrm{mm}}^{2}$]
[122]	2015	AVA with parasitic elliptical patch in the slot aperture	2–30	2–6	140×66×1.5
[15]	2017	AVA with fern fractal leaf-inspired structure	1.3–20	0–9	50.8×62×0.8
[48]	2017	AVA with multi-layer planar dieletric lens (AVA- MPDL)	1.3–30	2–16	171.8×149.8×1
[87]	2017	AVA with split-edge technique and half-elliptical-shaped dielectric lens	1–30	2.2–11	100×71.46×0.508
[134]	2020	Vivaldi with exponentially tapered slot, corrugations and grating elements inserted at the opening end of the exponential slot	1.9–6	5–8	128×70×0.8
[135]	2018	Flexible corrugated Vivaldi antenna	2–8	2–9	58.9×48×0.25
[136]	2019	AVA with square dieletric lens	2–10	4.62–13.01	75×140×1.6
[43]	2016	Elliptically tapered AVA with trapezoid-shaped dielectric lens	3.4–40	8–14	40×90×0.5
[47]	2014	Dielectric lens BAVA with low-cross-polarization	3–18	2.8–12	96×50×3.15
[50]	2010	BAVA including a director with high- permittivity material in the aperture	2.4–18	–	80×44×9.2
[82]	2015	AVA with elliptical strip conductors and two pairs of slots and circularly shaped loads	1.32–17	3.5–9.3	90×93.5×0.8
